# Acute distal biceps tendon rupture repair comparing single versus double-incision technique: A retrospective study with follow-up

**DOI:** 10.1177/17585732251352745

**Published:** 2025-07-07

**Authors:** Gard Kallhovd, Stein Atle Lie, Johannes Cornelis Schrama, Pål Høvding, Yngvar Krukhaug

**Affiliations:** 1 Faculty of Medicine, University of Bergen, Bergen, Norway; 2 Department of Orthopaedic Surgery, Haukeland University Hospital, Bergen, Norway

**Keywords:** Acute distal biceps tendon rupture repair, single incision, double incision, follow-up, smoking history

## Abstract

**Background:**

Single-incision (SI) and double-incision (DI) techniques are used for acute distal biceps tendon rupture repair. The purpose of this retrospective cohort study with follow-up was to examine if there is a difference between the techniques on early- and long-term outcomes.

**Methods:**

Hospital records from Haukeland University Hospital, Norway, (2007–2017) involving acute distal biceps tendon rupture repair matching inclusion criteria were analysed. Follow-up included assessing symptomatic and functional outcome, quality-of-life outcome (QuickDASH and EQ-5D), visual assessment scale (pain), and subjective health score. A smoking history was obtained.

**Results:**

We included 102 elbows in 100 patients, 99 males. Overall early complication rate was higher for the SI technique compared to the DI technique (25/43 vs. 11/58; *p* < 0.001). Long-term complications showed no statistically significant difference between the SI and DI technique (12/43 vs. 8/58; *p* = 0.078). The pronation range of motion favoured the SI technique compared to the DI technique (89.3° vs. 85.1°; *p* = 0.014). Supination strength favoured the DI technique compared to the SI technique (98.7 vs. 94.5; *p* = 0.030). Supination strength favoured non-smokers compared to former smokers (99.5 vs. 93.2; *p* = 0.009). The two techniques had similar quality-of-life outcomes.

**Conclusion:**

The DI technique has a lower risk of short-term complications. Both techniques have comparable symptomatic, functional, and quality-of-life long-term outcomes.

## Introduction

Rupture of the distal biceps tendon occurs most frequently in middle-aged men typically from 35 to 54 years of age (mean 46 years).^1^ This injury is most frequently caused by a forceful rapid eccentric contraction exceeding the tensile strength of the distal biceps tendon.^2^ Early surgical intervention has been recommended due to favourable functional long-term outcomes of both strength and endurance in elbow supination and flexion of the forearm compared to conservative treatment.^3–5^ Two different incisional approaches are used today for acute distal biceps tendon rupture. The single-incision (SI) technique comprises either a longitudinal incision following the medial border of the brachioradialis^6^ or a transverse incision located over the radial tuberosity.^7^ The other, is the double-incision (DI) technique using two small incisions, one transverse in the anterior cubital fossa and another at the dorso-radial aspect of the proximal forearm.^6^ After the introduction of the modified DI technique^8^ and various fixation techniques including suture anchors,^9^ Bio-Tenodesis screws^10,11^ and Endobutton,^12,13^ all involving the SI approach, success- and complication rates appear similar between the two techniques.^14^ Even though the two approaches have similar complication rates, certain complications occur more frequently with each of the surgical methods. Lateral antebrachial cutaneous nerve (LACN) apraxia occurs more often with the SI technique. While heterotopic ossification (HO) occurs more often with the DI technique.^15^

Recent research has focused on improving incisional placement to reduce to risk of complications, such as PIN injury when using the DI technique.^16^ Several studies have also been conducted on optimizing fixation techniques,^17^ some focusing on anatomic footprints of the short and long head of the biceps tendon.^18,19^ Anatomic compared to nonanatomic repair seems to be more efficient in producing supination, but only when the elbow is in 90° flexion.^20^

As of now, due to similar success and complication rates choosing between the SI and DI techniques depends on the surgeon and their preferences.^21,22^ The purpose of this study was to examine any difference between the two techniques on early- and long-term outcomes related to complications, symptoms, function and quality-of-life. Our hypothesis was that there was no statistically significant difference between the two incisional techniques. This study includes a large sample size with a high number of attending elbows at follow-up. Long-term quality-of-life outcome was extensively examined in comparison with earlier similar research.^21–28^

## Material and methods

### Haukeland University Hospital records

This is a retrospective cohort study with follow-up. Approval of this study came from the regional committees for medical and health research ethics in Norway (2018-004047-23). All consent-competent patients between 18 and 70 years operated for acute complete distal biceps tendon rupture at Haukeland University Hospital in Norway between 2007- and 2017 were included in this study. Patients suffering from injuries in the same extremity before or after the operation were excluded. Patients were identified by name and national identity number in the hospital records. Before inclusion, all patients were informed and consented to be part of the study. Hospital records contained details of the injury, surgery and early- and long-term complications. Early complications were defined as occurring within eight weeks after surgery and lasting no longer than two years. Long-term complications were defined as being related to the surgery and lasting longer than two years. All surgeries included using the SI approach were performed using a longitudinal incision. Prior to the introduction of Juggerknot anchors in 2009, Mitek anchors were used. From 2010, both Mitek- and JuggerKnot anchors were used interchangeably between all included SI and DI surgical procedures according to manufacturer recommendation. A total of eight surgeon consultants performed all included surgeries. Patients did not routinely receive prophylaxis for HO. All patients underwent similar post-operative care and rehabilitation.

### Follow-up

All patients were assessed during physical attendance at follow-up. Neurapraxias were assessed by examining all motor and sensory innervations from the elbow and distally. Assessing range of motion (ROM) of the elbow and hand comprised four subcategories: extension, flexion, supination, and pronation. These were compared with the non-injured extremity (except for bilateral injury) and measured using a goniometer. Extension and flexion were measured with the outstretched arm registered as 0. Pronation and supination were measured with the shoulder in a neutral position and the elbow at a 90° angle. Clinically relevant HO was verified by radiography. Clinically relevant HO was defined as associated symptoms or concerns affecting the patients’ well-being or daily function by the patient or clinician. Strength was assessed by isokinetic supination. The clinician measured supination strength in the injured extremity using the non-injured extremity as a point of reference (except with bilateral injury). Supination strength was defined in units from 0% to 100%. Non-injured extremity supination strength was determined as 100%. A score of 0 was determined as paralysis. Supination strength of the injured extremity was assessed by clinician expertise using force application to discern differences in relative supination strength between extremities. Three orthopaedic surgeons (YK, JS and PH) with >20 years’ experience each conducted the assessments.

Patient outcome was assessed using four different assessment tools. First, a shortened version of the Disability of Arm, Shoulder, and Hand: QuickDASH (Norwegian),^29^ measuring symptoms and function of the upper extremity when there is a musculoskeletal disorder of the upper limb. Second, EQ-5D measures quality-of-life through five dimensions: mobility, self-care, activities of daily life, pain/ discomfort, and depression/ anxiety. Third, a visual assessment score (VAS) from 0 to 10 was used to assess subjective pain on its’ own. Fourth, subjective health status was assessed by the patient from 0 to 100, 100 being in perfect health. Furthermore, smoking history was obtained, and grouped by non-smokers, former smokers, and smokers.

### Statistical analysis

Time intervals, ROM, supination strength, and the EQ-5D were analysed using student *T*-tests, except for comparing smoking history on EQ-5D where ANOVA was used. Pearson chi-square was used to analyse differences in early complication rates and long-term complication rates between the two groups. However, when comparing neural and non-neural complications Fisher's exact test was used due to few observations. Pearson chi-square was also used to compare educational level, group size differences between time periods and the three groups of smoking. QuickDASH, VAS, and subjective health status were analysed using the Mann-Whitney *U*-test.

## Results

See Table 1.

**Table 1.** Descriptive statistics for single-incision (SI) and double-incision (DI).

	SI (SD)	DI (SD)	*p*-value
Mean age (SD), time of injury	50.3 (8.8)	49.1 (8.4)	0.48
Mean age (SD), time of follow-up	55.4 (8.9)	57.0 (9.7)	0.43
Male gender	42/43	57/57	–
Dominant arm^a^	23/40	28/46	–
Bilateral (*N*)	1/43	1/57	–
Injury to surgery time days (SD)	9.9 (9.4)	7.0 (7.7)	0.094
Surgery to follow-up time years (SD)	4.82 (2.1)	7.81 (3.3)	<0.001
>9 years education^a^	39/41	42/45	0.72
University exam^a^	19/41	24/45	0.52

SD: standard deviation.

Continuous variables given as mean values with corresponding standard deviation. Frequencies given with denominators.

^a^ Registered at follow-up.

### Epidemiology

We included 102 elbows across 100 patients (99 males) in this study. Forty-four elbows received operation by SI and 58 by DI. The mean age at the time of injury was 50.3 and 49.1 in the SI and DI groups, respectively. In one patient in the SI group information about surgical complications was missing. The time between injury and surgery was not statistically significant different between the two groups (*p* = 0.094).

We found a statistically significant increase in surgical procedures in the SI group compared to the DI group for the last 4 years (35/44 vs. 22/58; *p* < 0.001). The increase was due to surgeon preference. However, we did not find a statistically significant difference between complication rates (*p* = 0.56) comparing the two time periods or the rate of long-term complications (*p* = 0.14).

### Early complications

**Figure 1.**
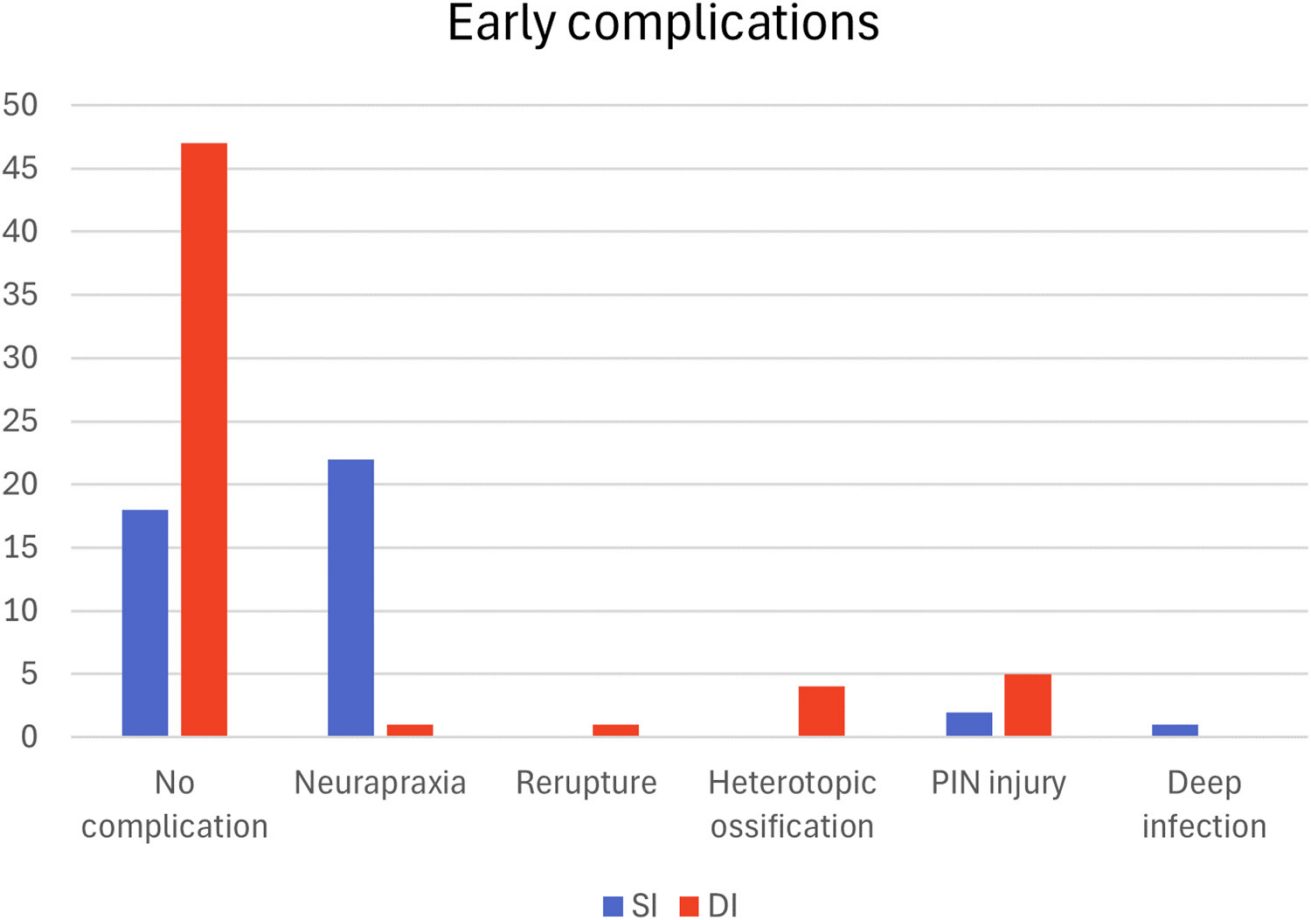
Early complications.

The SI group had a significant higher overall early complication rate, 25/43 (58%) compared to 11/58 (19%) in the DI group (*p* < 0.001). Subanalysis for the time period 2010–2017 was done due to two interchangeable anchoring methods reaching similar results (*p* = 0.002). Complications in the SI group were mostly due to neurapraxias, 24/25 cases, of these there were 14 LACN and two PIN. Five of 14 LACN were present at follow-up, a resolution of almost two-thirds of early LACN. We also identified one case with deep infection in the SI group. In the DI group, there were five PIN injuries (8.6%). We did not find a statistically significant difference in PIN injury between the two techniques (*p* = 0.44). All PIN injuries in both groups were resolved by the time follow-up took place. The DI group had four HO (6.9%), one re-rupture and one neurapraxia. There was a significant difference comparing neural and non-neural complications between the two techniques (*p* = 0.006) (Figure 1).

### Follow-up

Follow-up was done at a mean of 4.82 years and 7.81 years post-operatively (*p* < 0.001) for the SI and DI groups, respectively. Out of 102 elbows, 15 elbows were lost to follow-up. A total of 87 elbows (85 patients) attended follow-up. The mean age at follow-up was 55.4 and 57.0 in the SI and DI groups, respectively (*p* = 0.425). One patient in the SI group did not conduct the physical tests. Similarly in the DI group, one patient did not conduct the health questionnaire. Hence, 86 elbows assessed ROM, and supination strength and completed the health questionnaires (Table 2).

**Table 2.** Mean values (with SDs) for ROM and supination strength measured at clinical follow-up.

	Healthy, SI (SD)	Healthy, DI (SD)	*p*	Injured, SI (SD)	Injured, DI (SD)	*p*
*N*	38	44		40	46	
Flexion	124.2 (8.7)	122.7 (7.3)	0.40	123.4 (8.8)	122 (7.7)	0.428
Extension	0 (2.85)	0.91 (3.6)	0.22	−0.62 (3.8)	1.63 (7.5)	0.090
Supination	88 (11.9)	86 (9.3)	0.50	84.8 (15.0)	84.8 (9.7)	0.990
Pronation	89.5 (6.8)	86.4 (9.0)	0.08	89.3 (6.5)	85.1 (8.5)	0.014
Supination strength	–	–		94.5 (11.6)	98.7 (5.3)	0.030

SD: standard deviation; ROM: range of motion; SI: single-incision; DI: double-incision.

We found a statistically significant reduction in the pronation ROM in the DI group compared to the SI group (85.1 and 89.3, respectively; *p* = 0.014), not explained by differences in the healthy extremities (*p* = 0.084). Measuring ROM we found no statistically significant difference between flexion (*p* = 0.428), extension (*p* = 0.090) and supination (*p* = 0.990). Supination strength was significantly reduced in the SI group (94.5) compared to the DI group (98.7; *p* = 0.030).

In a total of 66 (77%) elbows we identified no long-term complications during follow-up. 12/40 (27.9%) patients in the SI group and 8/46 (13.8%) patients in the DI group had long-term complications at follow-up. This difference was not statistically significant (*p* = 0.078). Subanalysis for long-term complications in the time period 2010–2017 due to two interchangeable anchoring methods was not statistically significant with *p* = 0.47. We found a total of 12 complications in the SI group at follow-up, 93% were neurapraxias: five LACN, four radial sensory nerves, two radial motor nerves, and one HO. In the DI group, we found a total of eight complications during follow-up. Four HO (one was already treated surgically) of which two had reduced pronation to 60°, one LACN, one median motor nerve, and one median sensory nerve. Two cases of re-rupture were also identified in the DI group; however, these complications were not present at follow-up.

A smoking history was obtained during the follow-up. Forty elbows were non-smokers, 33 were former smokers, and 13 were smokers. Seventeen, 18, and six elbows were operated with the SI technique, respectively. We found no statistically significant difference in long-term complications when comparing non-smokers (10 elbows, 26%) and former smokers (five elbows, 15%; *p* = 0.275), non-smokers and smokers (five elbows, 38%; *p* = 0.377) and former smokers and smokers (*p* = 0.084). The two cases of re-rupture occurring before follow-up were both in the non-smoker group. Comparing ROM and supination strength, we only found a statistically significant difference between groups when looking at supination strength for non-smokers (99.5) and former smokers (93.2; *p* = 0.009), smokers measured 97.3.

Measuring daily function and quality of life, one patient in the DI group was not cognitively able to complete the screening. We found no statistically significant difference in QuickDASH (SI: 8.0; DI: 6.7; *p* = 0.337), VAS pain score (SI: 1.10; DI: 1.05; *p* = 0.446 (one patient in each group scored 7 and 8 points)), EQ-5D (*p* = 0.85) or health status (SI: 77.2; DI: 79.6; *p* = 0.121). Smoking history had no impact on EQ-5D when comparing smoking groups (*p* = 0.78).

**Figure 2.**
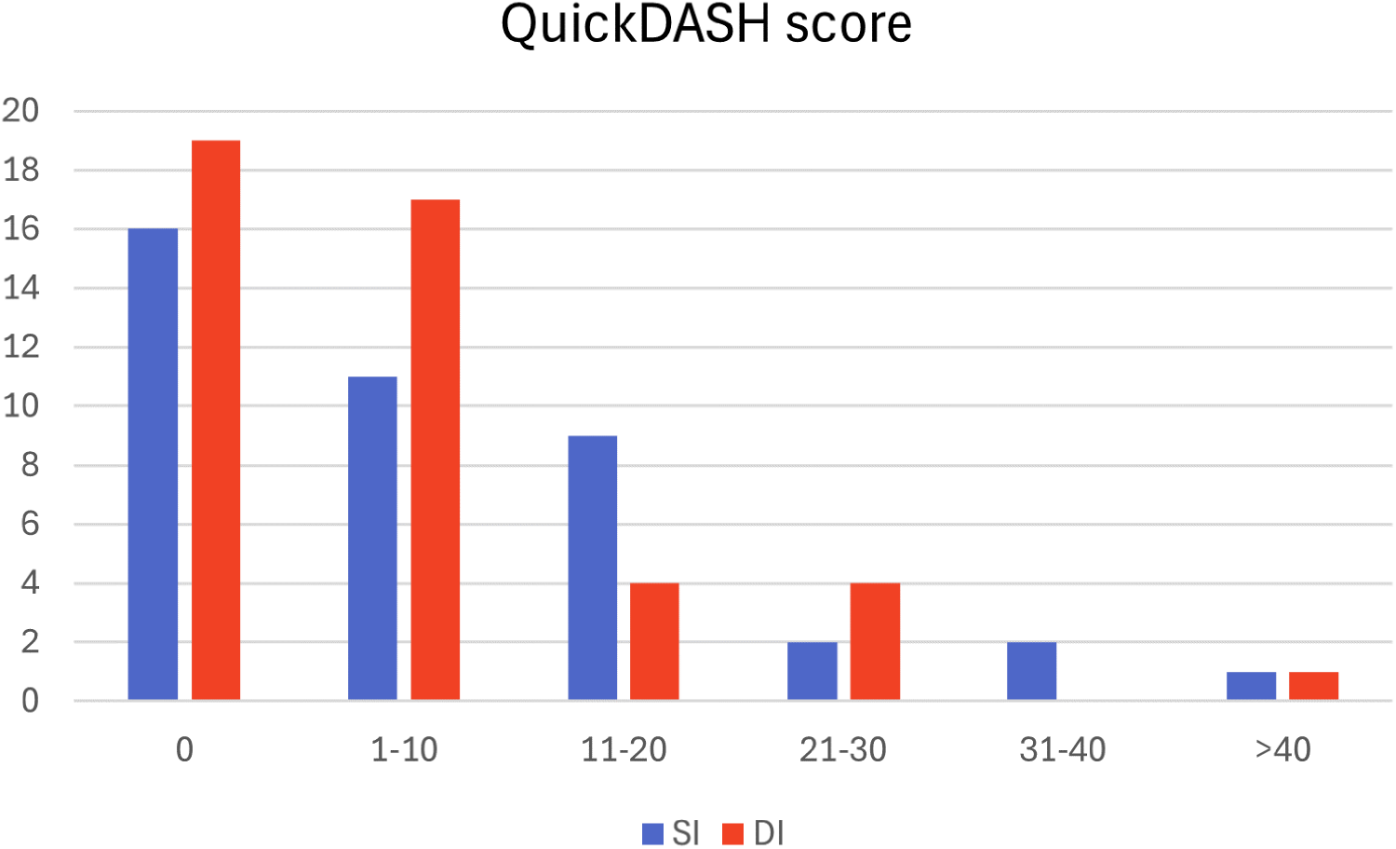
Patient-reported Disability of Arm, Shoulder, and Hand (QuickDASH).

During follow-up 74 elbows were working, 10 elbows were retired from work and two elbows were unemployed. Reasons for unemployment were unrelated to the rupture of the distal biceps tendon and repair (Table 3 and Figure 2).

**Table 3.** Mean values for health-related outcomes.

	SI (SD)	DI (SD)	*p*
*N*	41	45	
QuickDASH	8.0 (10.8)	6.7 (12.1)	0.337
VAS	1.10 (1.9)	1.05 (2.7)	0.446
EQ-5D	0.93 (0.07)	0.93 (0.09)	0.850
Health status	77.2 (14.5)	79.6 (21.3)	0.121

SD: standard deviation; SI: single-incision; DI: double-incision; QuickDASH: Disability of Arm, Shoulder, and Hand; VAS: visual assessment score; EQ-5D: EuroQol five-dimension.

## Discussion

To our knowledge, this is the largest retrospective cohort study with follow-up comparing the SI and DI techniques for the repair of acute distal biceps tendon rupture.

Earlier retrospective studies comparing the two incisional techniques have reached divergent results. Hogea et al.^23^ concluded that the SI technique yielded better results for functional outcome, patient satisfaction and lower risk of complication. On the other hand, several studies^24–26^ concluded with a lower post-operative complication rate using the DI technique compared to SI. Furthermore, functional outcome and patient satisfaction were comparable between the two incisional approaches during follow-up.^21,24^

We found a higher overall early complication rate with the SI technique constituting almost entirely neurapraxias. This finding is consistent with earlier clinical studies.^22,27^ In the DI group, clinically relevant HO and PIN injuries were the most frequent early complications in our study. A meta-analysis by Castioni et al.^14^ supports HO being the predominant complication found with the DI technique. However, there are divergent results regarding PIN injuries. In our study, we could not find a statistically significant difference between the techniques, which is supported by the study of Ford et al.,^25^ but in the study of Dunphy et al.,^26^ they did find a statistically significant difference in PIN injuries between the techniques. Due to conflicting results, further larger studies are necessary to elucidate which of the incisional methods have a greater risk of PIN injury if there truly is a difference.

We found a statistically significant difference in the number of SI procedures between the first period and the last 4 years of the research period compared to the DI group. This was due to surgeon preference. However, we did not find any statistically significant difference in early or long-term complications between the two time periods. Thus, we conclude that complications were due to different incisional techniques and not due to different practices or surgeon skills between the two time periods at the hospital.

The difference in complication rates between the two groups was not present during follow-up when comparing long-term complications. This finding is consistent with earlier research.^22^ LACN and radial sensory neurapraxia were the most frequent long-term complications in the SI group. This is consistent with the research by Shields et al.^30^ However, in their study the follow-up time was on average 5 months. In the study of Grewal et al.,^22^ they identified six long-term complications related to LACN in their follow-up at 6 months which was reduced to two at 24 months follow-up.

The most frequent long-term complication in the DI group was HO. There was one case in the DI group that developed HO and he had been treated with new surgery before the follow-up. Thus, this case was not registered as a long-term complication. In our study, the complication rates of HO are similar to findings in earlier studies.^15^ However, a bit higher than Grewal et al.^22^ One possible explanation could be their use of indomethacin as prophylaxis against HO.

ROM tests showed no statistically significant difference between groups when looking at flexion which is contrary to earlier clinical findings.^14,27,30^ In their studies, flexion was in favour of the SI technique. Furthermore, the same studies support our finding of no statistically significant difference between the groups related to extension and supination.

We found a statistically significant difference between the groups when looking at pronation and supination strength. Pronation was superior in the SI group while supination strength was superior in the DI group. These results are aligned with the research of Grewal et al.^22^ However, they are inconsistent with the research done by El-Hawary et al.^27^ and Shields et al.^30^ both showing no statistically significant difference in pronation and supination strength. Our results, even if statistically significant, might not have clinical implications for each one specific patient due to small differences.

Safran and Graham^2^ revealed in their findings an increased risk of 7.5 for distal biceps tendon rupture for smokers compared to non-smokers. Similarly, smoking has been reported to have an increased risk of re-rupture^28^ and non-home discharge.^31^ Our results do not support smoking having an increased risk of re-ruptures. To the best of our knowledge, this study is the first to compare smoking history and long-term functional outcomes during follow-up after acute distal biceps tendon repair. Our study found no statistically significant difference in ROM between the groups, but we did find a statistically significant difference in supination strength between smokers and former smokers. EQ-5D was not different between the groups. Thus, our conclusion is in accordance with our findings; that differences in smoking history have minimal impact on long-term functional outcomes. Further research is needed to support this claim.

We found no statistically significant difference between the two incisional techniques when comparing QuickDASH, which supports the findings in earlier research.^14,24^ A Norwegian normative population-based study by Aasheim and Finsen^32^ identified the mean QuickDASH score for men aged 50–59 to be 12 (confidence interval: 9–15). Thus, long-term results on QuickDASH of the two incisional techniques do not seem to have a negative impact on QuickDASH score when compared to the general population.

Pain VAS was not statistically significantly different between the two groups. This is supported by earlier research.^22,30^ Furthermore, even though the mean pain VAS score was low in both groups there were a few who acquired aggravating pain. Thus, the techniques are not without risk of debilitating pain, even though the risk is low.

A Norwegian normative population-based study on EQ-5D-5L determined the mean score for men aged 50–59 to be 0.767 and the mean score for the whole population below upper secondary school (94% of this study's patients has completed lower secondary school) to be 0.724. Completing <4 years of higher education resulted in a mean EQ-5D of 0.844 (50% of the patients in this study have completed at least one university exam).^33^ We would thus conclude that long-term outcomes on either incisional technique do not have a negative impact on EQ-5D compared to the general population with corresponding age. Subjective health status was not statistically significantly different between the two groups, understating the favourable long-term outcome of the two techniques.

## Strengths and limitations of the study

This study has a considerable number of operated elbows, and the number of elbows attending the follow-up is high. Furthermore, this study is the first to examine long-term outcomes in relation to smoking history when comparing the SI and DI techniques. We also used several questionnaires assessing slightly different aspects of symptomatic, functional and quality-of-life outcomes, generating a more comprehensive understanding of the patient's well-being.

A total of 15 elbows could not attend follow-up. The drop-out rate was higher for the DI group compared to the SI group. This difference could have affected the results on long-term complication outcome between the groups, if patients not attending were due to satisfying results. The patients’ personal reasons for dropping out of the study are not known to the authors.

Supination strength was assessed by clinicians’ expertise measuring differences between the injured and non-injured extremities. Obtaining the differences in strength through objective measurements using appropriate instruments would certainly give more accurate results. However, measuring absolute supination strength with quantitative difference in extremity was not regarded among this papers’ aims, but rather clinical and subjective perceived functional outcome for the patient.

## Conclusion

Our data suggests that using the SI technique has a higher risk of complications, albeit mostly short-term. The DI technique has a lower rate of complications, but the complications which do arise do not seem to dissipate at the same rate as in the SI group. Long-term functional outcomes are comparable between the two techniques, on which smoking habits seem to have minimal impact. Both approaches have good long-term outcomes on well-being, however, a few patients might experience debilitating pain. The long-term risk for affecting well-being is low for both surgical approaches.

Our study finds the DI technique to be superior short-term. Further larger studies, preferably randomized controlled trials with national collaboration between hospitals due to low incident numbers are needed to elucidate possible differences.
